# Young Swimmers' Anthropometrics, Biomechanics, Energetics, and Efficiency as Underlying Performance Factors: A Systematic Narrative Review

**DOI:** 10.3389/fphys.2021.691919

**Published:** 2021-09-16

**Authors:** Jorge E. Morais, Tiago M. Barbosa, Pedro Forte, António J. Silva, Daniel A. Marinho

**Affiliations:** ^1^Department of Sport Sciences, Instituto Politécnico de Bragança, Bragança, Portugal; ^2^Research Center in Sports Health and Human Development (CIDESD), University of Beira Interior, Covilhã, Portugal; ^3^Department of Sports, Higher Institute of Educational Sciences of the Douro, Penafiel, Portugal; ^4^Department of Sport Sciences, University of Trás-os-Montes and Alto Douro, Vila Real, Portugal; ^5^Department of Sport Sciences, University of Beira Interior, Covilhã, Portugal

**Keywords:** talent, identification, development, swimming, determinants, sports career

## Abstract

**Introduction:** In youth swimming, researchers are interested in understanding how anthropometry and parameters related to swimming technique (biomechanics, energetics, and efficiency) influence the performance. However, there is not any review in the literature that consolidates the body of knowledge of this topic. The objective of this study was to review systematically the current body of work on the influence of determinant factors related to swimming technique (biomechanics, energetics, and efficiency) and anthropometry in the young performance of swimmers.

**Methods:** The Preferred Reporting Items for Systematic Reviews and Meta-Analyses (PRISMA) guidelines were used to identify relevant studies.

**Results:** After screening, 240 studies were analyzed and 59 related to swimming performance, and its determinant factors were retained for synthesis. Studies revealed a high-quality index by PEDro scale (mean score was 7.17 ± 1.40). Twenty-five studies were longitudinal designs and the remaining 34 cross-sectional designs. Most of the studies (*N* = 39, 66.1%) reported concurrently two or more determinant factors (anthropometrics, biomechanics, energetics, and efficiency).

**Conclusion:** Youth swimming research relies on a multifactorial assessment. From the synthesis, it is possible to conclude that the performance of young swimmers is characterized by a multifactorial, holistic, and dynamic phenomenon. Better performance has always been related to better swimming technique and higher anthropometrics. This suggests that both anthropometrics (i.e., nature) and training (i.e., nurture) play key roles in the swimming performance of young swimmers.

## Introduction

One of the major topics of interest in sports science is the identification of talented young athletes. This process is based on talent identification and development (TID) programs that aim to identify young athletes with potential for success in adult/elite sport (Blume and Wolfarth, 2019). Detecting talent at an early stage is considered a key factor in increasing a chance of a country of achieving success in sports (Vaeyens et al., 2009). Competitive swimming is one of the three main modern Olympic sports. In competitive swimming, Olympic, and World records are broken on a regular basis, challenging the limits of athletes. Practitioners and researchers are eager to predict the next top-ranked swimmer who will contribute to the superiority of their country at major international competitions.

Talent identification and development programs follow standard steps: (1) identifying the athletes with the potential to deliver the best performances in adulthood and determining the variables responsible for such performances; (2) understanding the development and changes in performance and its determinant factors, according to a training program, and; (3) following up in order to allow to understand the variation of such variables and its relationship with performance over a given time (Morais et al., 2017). To get deeper insights into how determinant factors of swimmers change over time, their interaction and their effect on performance, researchers, and coaches should focus on a long-term approach (Staub et al., 2020a; Zacca et al., 2020). Long-term athlete development (LTAD) programs focus on providing young athletes with fundamental motor skills in tandem to their maturation stage (Martindale et al., 2005; Lang and Light, 2010).

Literature reports that performance in youth swimming is highly dependent on variables related to technique (i.e., nurture) and body dimensions (i.e., nature) (Abbott et al., 2021). Thus, research on young swimmers has been largely focused on the assessment of anthropometrics (Geladas et al., 2005; Nevill et al., 2020), strength and conditioning (Garrido et al., 2010b; Amaro et al., 2017), biomechanics (Morais et al., 2012; Silva et al., 2012), energetics, and efficiency (Denadai et al., 2000; Toubekis et al., 2006), as well as interactions among some or all of them (Morais et al., 2017; Barbosa et al., 2019). Nonetheless, most of these are cross-sectional designs. Such research design does not provide substantial information on the dynamic and complex interactions among the performance determinants over time (Morais et al., 2017). Conversely, longitudinal designs can help gather information on: (1) how determinant factors interplay and affect performance; (2) the dynamic changes that take place at these early ages, and; (3) the change of the partial contribution of each determinant factor in the performance over time (Lätt et al., 2009a,b; Morais et al., 2014a). Notwithstanding, in the last decade, it has been suggested that research on sports performance should adopt a multidisciplinary approach to better understand the athlete (Phillips et al., 2010; Seifert et al., 2013). Moreover, the relationship with the environment must be taken into account, as this relationship is considered under a complex and dynamic system framework (Phillips et al., 2010; Seifert et al., 2013). If so, it will be possible to understand the partial contribution of each determinant factor or set of factors in the performance, which will most likely change over time, as aforementioned (Barbosa et al., 2014; Morais et al., 2015).

Literature reports a review study about the relationship between performance and determinant factors in master swimmers (Ferreira et al., 2016). More recently, Koopmann et al. (2020) have systematically reviewed technical skills in talented youth athletes (which included three articles about swimmers). That said, there is no review that consolidates the available evidence of how different determinant factors can affect youth swimming performance. Therefore, the aim of this study was to review the current body of work on the influence of determinant factors related to swimming technique (biomechanics, energetics, and efficiency) and anthropometrics in the performance of young swimmers.

## Methods

### Literature Search and Article Selection

The Web of Science, PubMed, and Scopus databases were searched to identify studies that aimed to identify, analyze, or predict the performance of young swimmers and its determinant factors (anthropometrics, biomechanics, energetics, and efficiency). These electronic search databases were chosen because they are the most used in sports science. As an initial search strategy, the title, abstract, and the studies keywords were identified and read carefully for a first scan and selection of the journal articles. To search the articles, the following fields were used: (1) Web of Science—“Topic”; (2) PubMed—“All fields”; and (3) Scopus—“Article title, abstract, keywords.” A Boolean search strategy was used with the operators AND, OR, and a combination of the keywords presented in Table 1 (whenever suitable). If one of these fields (title, abstract, and keywords) was not clear about the topic under analysis, the complete article was read and fully reviewed to ensure its inclusion or exclusion. After deleting all duplicated and unrelated articles, 59 articles were included. The final search was carried out on March 21, 2021. Table 1 presents the used PI(E)CO search strategy (P—patient, problem or population; I—intervention; E—exposure; C—comparison, control, or comparator; O—outcomes).

**Table 1:** PI(E) CO (P—patient, problem or population; I—intervention; E—exposure; C—comparison, control, or comparator; O—outcomes) search strategy.

**Population**	**Intervention or Exposure**	**Comparison (design)**	**Outcome**
Swimmer*	Talent	Cross-sectional	Performance
Athlete*	Identification	Longitudinal	Velocity/speed
Youth	Development	Experimental	Length
Child*	Long-term development	Exploratory	Area
Boy*	Anthropometrics	Descriptive	Volume
Girl*	Biomechanics	Randomized control trial	Mass
Young	Energetics		Girth
Age-group*	Efficiency		Skinfold
	Motor control		Stroke length
	Strength and conditioning		Stroke frequency
			Stroke rate
			Intra-cyclic variation of velocity/speed
			Passive drag
			Active drag
			Coefficient of drag
			Oxygen uptake
			Oxygen consumption
			Lactate
			Heart rate
			Aerobics
			Anaerobic lactic
			Anaerobic alactic
			Energy cost
			Energy expenditure
			Propelling efficiency
			Froude efficiency
			Stroke Index
			Critical velocity/speed
			Index of coordination
			Strength
			Maximal strength
			Power
			Mechanical power

*Truncation to retrieve words with different endings.

The inclusion criteria were the following: (1) written in English; (2) published in a peer-reviewed journal; (3) related to assessment of the performance of young swimmers (i.e., race events or swim trials/bouts) and its determinant factors (anthropometrics, biomechanics, energetics, and efficiency); (4) included healthy and able-bodied swimmers, and; (5) reported an average sample age limited to the age of 13 (it is considered that children tend to enter the puberty stage from this age onwards—Mirwald et al., 2002). The exclusion criteria were: (1) studies that included disabled swimmers or with any pathology; (2) review papers, conference papers, and books; (3) studies including animal models; (4) publications not related to the topic in question (e.g., in other scientific fields, such as nutrition, psychology, or any other topic not related to performance); (5) studies that recruited several age groups, but did not clearly report the average of at least an age group of 13 years or under.

**Figure 1:**
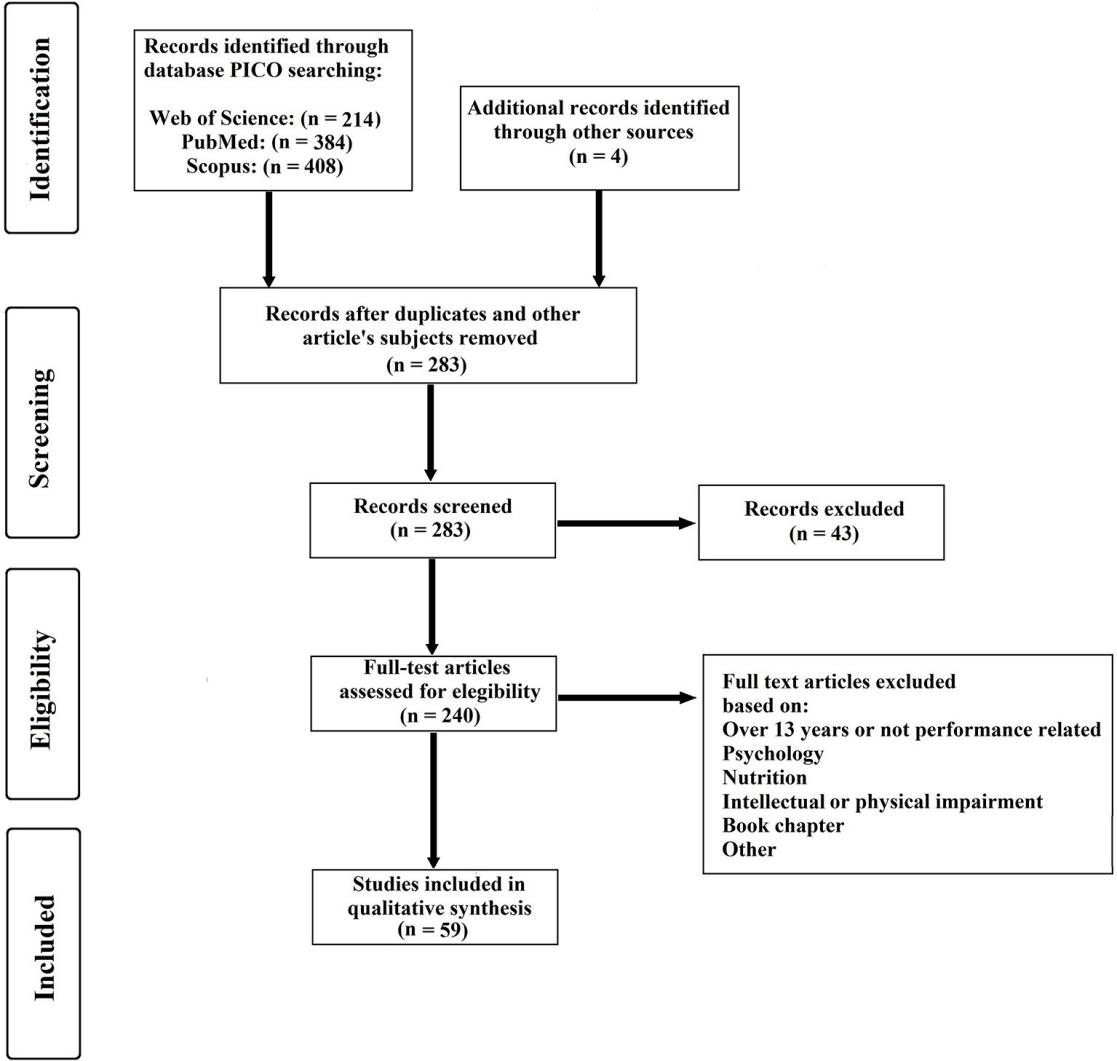
Summary of PRISMA flow for search strategy.

Figure 1 depicts the PRISMA flow diagram for identifying, screening, checking eligibility, and inclusion of the articles. There were four articles (Figure 1—“Additional records identified through other sources” that were obtained by submissions reviewed and based on references from the articles retained.

### Quality Assessment

The PEDro scale was used to assess the quality of the selected articles. It was observed that this scale is a suitable and valid tool to assess the methodological quality (de Morton, 2009). Two reviewers read all the included articles and scored them according to the scale items (poor quality if score ≤ 3; fair quality if the score is between 4 and 5; high quality if the score is between 6 and 10) (de Morton, 2009). Afterwards, the Cohen's Kappa (K) was computed to assess the agreement between reviewers. It was interpreted as: (1) no agreement if *K* ≤ 0; (2) none to slight agreement if.01 < *K* ≤ 0.20; (3) fair if.21 < *K* ≤ 0.40; (4) moderate if.41 < *K* ≤ 0.60; (5) substantial if.61 < *K* ≤ 0.80, and; (6) almost perfect if.81 < *K* ≤ 1.00. Studies were compared based on the: (1) research design (cross-sectional vs. longitudinal designs), and (2) year of publication (published before or in 2010 vs. published after 2010). In both comparisons, distribution was non-normal. Thus, the Mann–Whitney U test (*p* ≤ 0.05) was selected for further inferential analysis.

## Results

PEDro mean score was 7.17 ± 1.40 points (i.e., high quality). The Cohen's Kappa yielded an almost perfect agreement between reviewers (*K* = 0.937, *p* < 0.001). There were non-significant differences in PEDRo scores based on research design (*p* = 0.651), or year of publication (*p* = 0.477).

Table 2 summarizes the sample demographics, including the sample size, chronological age, maturation stage, years of experience, and competitive level based on FINA points.

**Table 2:** The summary of the sample demographics of each study included for analysis.

**Source**	**Sample**	**Tanner stage**	**Years of experience**	**Pool length**	**Race/trial event**	**FINA points**
Abbes et al. (2018)	*n* = 14 boys: 13.00 ± 2.00 years	n.a.	At least 4 years	50 m	50 m Freestyle	520.00 ± 98.00
Abbes et al. (2020)	*n* = 17 boys: 13.00 ± 2.00 years	n.a.	At least 4 years	50 m	50 m Freestyle	520.00 ± 98.00
Abbott et al. (2021)	*n* = 48 boys (between 10 and 13 years)	Maturity status (years pre/post peak height velocity): between −2.4 ± 0.29 and 0.2 ± 0.46	n.a.	50 m	200 m Freestyle	
Alshdokhi et al. (2020)	*n* = 28 boys: 12.60 ± 2.60 years	n.a.	n.a.	25 m	50 m and 100 m Freestyle, Backstroke	n.a.
Amaro et al. (2017)	*n* = 21 boys: 12.70 ± 0.80 years	2.10 ± 0.40	At least 2 years	25 m	50 m Freestyle	n.a.
Barbosa et al. (2010)	*n* = 38 boys: 12.53 ± 0.58 years	1–2	n.a.	25 m	200 m Freestyle	n.a.
Barbosa et al. (2014)	*n* = 34 girls and 33 boys: 12.83 ± 1.26 years	1–2	At least four years	25 m	100 m Freestyle	n.a.
Barbosa et al. (2015)	*n* = 49 boys: 12.51 ± 0.77 years; 51 girls: 12.24 ± 0.71 years	1–2	n.a.	25 m	100 m Freestyle	n.a.
Barbosa et al. (2019)	*n* = 75 boys: 11–13 years	1–2	At least two years	n.a.	100 m Freestyle	n.a.
Bielec and Jurak (2019)	*n* = 26 boys: 12.10 ± 0.50 years; 15 girls: 12.20 ± 0.50 years	Boys: 1.80 ± 0.60 Girls: 2.10 ± 0.70	2.40 ± 0.50	25 m	50 m Freestyle, and 200 m Individual Medley	Boys 50 m: 202.00 ± 64.40 Girls 50 m: 279.20 ± 58.30 Boys 200 m: 211.50 ± 55.90 Girls 200 m: 280.60 ± 46.40
Costa et al. (2011)	*n* = 242 boys	n.a.	n.a.	n.a.	50 m, 100 m, 200m, 400 m, 800 m, and 1500 m Freestyle	n.a.
de Mello Vitor and Böhme (2010)	*n* = 24 boys: 13.00 ± 0.70 years	3–4	3 to 4 years	50 m	100 m Freestyle	n.a.
Denadai et al. (2000)	Beginners: *n* = 4 boys and 6 girls: 11.20 ± 0.90 years Trained: *n* = 3 boys and 3 girls: 11.10 ± 0.90 years	n.a.	Beginners: 1–2 years; Trained: 3–5 years	25 m	50 m, 100 m, and 200 m Freestyle	n.a.
Duché et al. (1993)	*n* = 25 boys: 11.30 ± 1.00 years	1	2 years	n.a.	50 m, 100 m, 200m, and 400 m Freestyle	n.a.
Ferraz et al. (2020)	Under 12 level: *n* = 25 girls (12.48 ± 0.30 years); *n* = 24 boys (12.69 ± 0.26 years) Under 13 level: *n* = 23 girls (11.63 ± 0.28 years)	n.a.	n.a.	25 m	50 m, and 400 m Freestyle	n.a.
Ferreira et al. (2019)	n = 14 boys: 11.90 ± 1.08 years; 29 girls: 10.74 ± 0.91 years	Boys: 2.93 ± 0.95 Girls: 2.71 ± 1.15	n.a.	25 m	400 m Freestyle	n.a.
Ferreira et al. (2021)	*n* = 24 boys: 12.51 ± 0.99 years; 10 girls: 11.24 ± 0.88 years	Boys: 2.94 ± 1.04 Girls: 3.05 ± 1.10	n.a.	n.a.	400 m Freestyle	n.a.
Figueiredo et al. (2016)	*n* = 51 boys and 52 girls: 11.80 ± 0.80 years	n.a.	n.a.	25 m	25 m Freestyle trial	n.a.
Garrido et al. (2010a)	*n* = 16 boys and 12 girls: 12.01 ± 0.56 years	1–2	n.a.	25 m	25 m and 50 m Freestyle	n.a.
Garrido et al. (2010b)	*n* = 14 boys and 11 girls: 12.08 ± 0.76 years	1–2	n.a.	25 m	25 m and 50 m Freestyle	n.a.
Geladas et al. (2005)	*n* = 178 boys: 12.78 ± 0.05 years; 85 girls: 12.68 ± 0.06 years	Boys' biological age: 14.17 ± 0.13 Girls' biological age: 13.47 ± 0.13	n.a.	50 m	100 m Freestyle	n.a.
Hue et al. (2013)	*n* = 61 boys and 65 girls: 12.00 ± 1.30 years	1–2	n.a.	50 m	400 m Freestyle	n.a.
Jürimäe et al. (2007)	*n* = 15 boys: 11.90 ± 0.30 years	1–2	3.00 ± 1.10	25 m	400 m Freestyle	n.a.
Kjendlie et al. (2004a)	*n* = 10 boys: 11.70 ± 0.80 years	n.a.	4.30 ± 1.40	25 m	50 m and 100 m Freestyle	n.a.
Kjendlie and Stallman (2008)	*n* = 9 boys: 11.70 ± 0.80 years	n.a.	n.a.	25 m	25 m Freestyle trial	n.a.
Lätt et al. (2009a)	*n* = 29 boys: 13.0 ± 1.80 years	2.30 ± 1.00	3.00 ± 1.10	25 m	400 m Freestyle	n.a.
Lätt et al. (2009b)	*n* = 26 girls: 12.70 ± 2.20 years	2.30 ± 0.80	3.70 ± 1.00	25 m	400 m Freestyle	n.a.
Majid et al. (2019)	*n* = 4 boys: 11.15 ± 0.96 years	n.a.	n.a.	50 m	50 m Breaststroke	n.a.
Marinho et al. (2011)	*n* = 12 boys and 8 girls: 12.10 ± 0.72 years	n.a.	3.70 ± 1.26	n.a.	50 m, 100 m, and 200 m Freestyle, Backstroke, Breaststroke, and Butterfly	n.a.
Marinho et al. (2020)	*n* = 75 boys and 76 girls: 13.02 ± 1.19 years	n.a.	3.36 ± 0.77 years	25 m	100 m Freestyle	n.a.
Mezzaroba and Machado (2014)	*n* = 13 boys: 10.70 ± 0.90 years; *n* = 11 boys: 13.00 ± 0.50 years	2.20 ± 0.80 and 3.60 ± 0.80	3.50 ± 1.90 and 5.70 ± 3.30 years	50 m	100 m, 200 m, and 400 m Freestyle	n.a.
Morais et al. (2012)	*n* = 73 boys: 12.72 ± 1.03 years; 64 girls: 11.47 ± 0.66 years	1–2	n.a.	25 m	100 m Freestyle	n.a.
Morais et al. (2013a)	*n* = 62 boys: 12.76 ± 0.72 years; 64 girls: 11.89 ± 0.93 years	1–2	n.a.	25 m	100 m Freestyle	n.a.
Morais et al. (2013b)	n = 15 boys: 12.30 ± 0.63 years; 18 girls: 11.77 ± 0.92 years	1–2	3.18 ± 0.52 years	25 m	100 m Freestyle	n.a.
Morais et al. (2014a)	*n* = 14 boys: 12.33 ± 0.65 years; 16 girls: 11.15 ± 0.55 years	1–2	3.40 ± 0.56 years	25 m	100 m Freestyle	Boys: 284.85 ± 67.48 Girls: 322.56 ± 45.18
Morais et al. (2014b)	*n* = 14 boys, 7 high skill: 12.83 ± 0.37 years, 7 average skill: 11.83 ± 0.37 years; 16 girls, 8 high skill: 11.42 ± 0.49 years, 8 average skill: 10.83 ± 0.37 years	1–2	3.40 ± 0.56 years	25 m	100 m Freestyle	Boys (high skill: 294.40 ± 40.00; average skill: 166.20 ± 17.50) Girls (high skill: 334.30 ± 39.50; average skill: 229.10 ± 33.90
Morais et al. (2015)	*n* = 15 boys: 12.30 ± 0.60 years; 18 girls: 11.70 ± 0.90 years	1–2	3.18 ± 0.52 years	25 m	100 m Freestyle	Boys: 227.90 ± 69.80 Girls: 291.10 ± 66.20
Morais et al. (2016)	*n* = 49 boys: 12.50 ± 0.76 years; 51 girls: 12.20 ± 0.71 years	1–2	3.10 ± 0.71 years	25 m	100 m Freestyle	n.a.
Morais et al. (2017)	*n* = 47 boys: 12.04 ± 0.81 years; 47 girls: 11.22 ± 0.98 years	n.a.	3.18 ± 0.62 years	25 m	100 m Freestyle	Boys: 217.70 ± 69.50 Girls: 277.70 ± 68.70
Morais et al. (2020a)	*n* = 22 boys: 12.79 ± 0.71 years; 32 girls: 11.78 ± 0.85 years	1–2	n.a.	25 m	100 m Freestyle	Boys: 297.58 ± 87.72 Girls: 330.35 ± 79.80
Morais et al. (2020b)	*n* = 14 boys: 12.70 ± 0.63 years; 16 girls: 11.72 ± 0.71 years	1–2	n.a.	25 m	100 m Freestyle	Boys: 234.86 ± 69.76 Girls: 288.75 ± 67.01
Moreira et al. (2014)	n = 12 boys: 12.80 ± 0.90 years; 13 girls: 12.00 ± 0.90 years	1–2	3.18 ± 0.52 years	n.a.	25 m Freestyle trial	n.a.
Nevill et al. (2020)	*n* = *n* = 39 boys: 11.50 ± 1.30 years; n = 20 girls: 12.10 ± 1.00 years; *n* = 13.00 ± 1.00 years	2.33 ± 1.10, 0.04 ± 1.00, 0.82 ± 0.96 maturity offset years	n.a.	n.a.	100 m Breaststroke and Backstroke	n.a.
Ozeker et al. (2020)	*n* = 15 girls: 11.18 ± 0.80 years; *n* = 15 girls: 11.16 ± 0.83 years	n.a.	At least 3 years	50 m	50 m and 400 m Freestyle	n.a.
Poujade et al. (2003)	*n* = 3 girls and 8 boys: 12.40 ± 0.50 years	n.a.	4–5 years	50 m	400 m Freestyle	n.a.
Poujade et al. (2002)	*n* = 3 girls and 8 boys: 12.40 ± 0.50 years	n.a.	5–6 years	n.a.	400 m Freestyle	n.a.
Saavedra et al. (2013)	*n* = 67 girls: 11.51 ± 0.55 years	n.a.	n.a.	n.a.	Best score according to the LEN table of competitive performance level	n.a.
Saavedra et al. (2010)	*n* = 67 girls: 11.50 ± 0.60 years	2.99 ± 1.19	n.a.	25 m	Fastest of three competitive events swum in one of the four strokesat any of four different race distances (i.e., 100 m, 200 m, 400 m, and 800 m)	n.a.
Sammoud et al. (2018)	*n* = 39 boys: 11.50 ± 1.30 years; 20 girls: 12.00 ± 1.00 years	Boys: −2.30 ± 1.10; girls: 0.04 ± 1.00 maturity offset years	n.a.	25 m	100 m Breaststroke	n.a.
Sammoud et al. (2019)	(*n* = 26 boys) two groups: 10.30 ± 0.40 and 10.50 ± 0.40 years	−3.10 ± 0.30 and −2.80 ± 0.30 years until peak height velocity	2.00 ± 1.60 years	50 m	15 m, 25 m, and 50 m Freestyle trial	n.a.
Sammoud et al. (2021)	(*n* = 22girls) two groups: 10.01 ± 0.57 and 10.50 ± 0.28 years	−1.50 ± 0.50 and −1.34 ± 0.51 maturity offset	2.00 ± 1.40 years	50 m	25 m, and 50 m Freestyle trial	n.a.
Seffrin et al. (2021)	*n* = 16 boys: 11.50 ± 0.52 years; 6 girls: 11.67 ± 0.52 years	n.a.	n.a.	n.a.	100 m and 400 m Freestyle	n.a.
Silva et al. (2012)	(*n* = 36 boys: 12.42 ± 0.08 years; and 24 girls: 11.08 ± 0.08 years)	Boys: 2–3 Girls: 2–3	3.75 ± 0.87 and 3.38 ± 0.77 years	n.a.	25 m Backstroke trial	n.a.
Silva et al. (2013)	Pubertal: n = 36 boys: 12.42 ± 0.08 years; 24 girls: 11.08 ± 0.08 years Post-pubertal: *n* = 20 boys: 12.65 ± 0.11 years; 34 girls: 11.71 ± 0.08 years	Pubertal: 1–2 Post-pubertal: 3–5	Pubertal boys: 3.75 ± 0.87 years; girls: 3.38 ± 0.77 years Post-pubertal boys: 3.75 ± 1.25 years; girls: 3.35 ± 1.07 years	n.a.	25 m Freestyle trial	n.a.
Staub et al. (2020b)	*n* = 952 boys and 936 girls: 11 years	n.a.	n.a.		50 m, 100 m, 200 m, 400 m Freestyle; 50 m, 100 m, 200 m Breaststroke and Backstroke; 50 m and 100 m Butterfly; 200 Individual Medley	Swimmers ranked at 18 years: 321.90 ± 75.20 Swimmers not ranked at 18 years: 313.80 ± 73.70
Staub et al. (2020a)	*n* = 842 boys and 863 girls: 11 years	n.a.	n.a.		50 m, 100 m, 200 m, and 400 m Freestyle; 50 m, 100 m, and 200 m for both Breaststroke and Backstroke; 50 m, and 100 m Butterfly; 200 m Individual Medley	Relationships between success at age 18 (1–1000 FINA points), to within-sport specialization and age of entry
Tijani et al. (2019)	*n* = 22 boys and 18 girls12.30 ± 0.56 years	n.a.	7.10 ± 0.50 years	25 m	50 m Freestyle	n.a.
Tsalis et al. (2012)	*n* = 8 girls: 10.40 ± 0.60 years	n.a.	n.a.	50 m	50 m, 100 m, 200 m, and 400 m Freestyle	n.a.
Zarzeczny et al. (2013)	*n* = 24 boys: 12.20 ± 0.10 years	n.a.	n.a.	25 m	50 m, and 400 m Freestyle and Breaststroke	n.a.

*n.a., not applicable (i.e., not reported)*.

Table 3 presents the summary of the studies purpose, research design, type of collected data (anthropometrics, biomechanics, energetics, and efficiency), and performance. Overall, swimming performance (time or speed) was clearly reported (normative data for time or speed at a given distance) in 51 reviewed studies (86.4%) (Table 3). Out of 59 included studies, 25 (42.4%) were based on longitudinal designs, and the remaining 34 (57.6%) were cross-sectional (Table 3). Fifty-four studies (91.5%) reported anthropometric parameters, including 34 cross-sectional designs and 20 longitudinal designs. Also, 54 studies (91.5%) analyzed the biomechanics (32 cross-sectional and 22 longitudinal designs), and 42 (71.2%) the energetics and efficiency (25 cross-sectional and 17 longitudinal designs) (Table 3). Thirty-nine studies (66.1%) reported anthropometrics, biomechanics, energetics and efficiency, and performance concurrently (i.e., interdisciplinary research). Three studies (5.1%) focused exclusively on tracking the swimming performance from childhood to adulthood.

**Table 3:** The summary of the purpose, design, type of data collected (anthropometrics, biomechanics, energetics/efficiency), and performance of the studies included.

						**Performance**	
**Source**	**Purpose**	**Design**	**Anthropometrics**	**Biomechanics**	**Energetics/Efficiency**	**Initial**	**Final**
Abbes et al. (2018)	To investigate whether tethered swimming before a 50 m freestyle swimming sprint could be an effective post-activation potentiation method to improve performance	Longitudinal	BM, H	CMJ, SL	RPE, Bl	50 Free CG: 32.48 ± 3.35 s 50 Free EG: 32.68 ± 3.68 s	
Abbes et al. (2020)	To investigate performance, biomechanical, physiological, and psychophysiological effects of a simple and easily organized post-activationpotentiation re-warm-up performed before a 50m freestyle swimming sprint	Longitudinal	BM, H	SF, SL	RPE, Bl, HR	50 Free Push-ups group: 32.62 ± 2.81 s 50 Free Squat jump group: 32.42 ± 2.32 s 50 Free Burpees group: 32.46 ± 2.26 s 50 Free CG: 32.84 ± 2.53 s	
Abbott et al. (2021)	To examine the longitudinal relationships between maturity status, technical skill indices, and performance in male youth competitiveswimmers. To determine whether individualdifferences in maturation influenced relationships between technicalskill level and swim performance.	Longitudinal (4 months)	BM, H	v	SI, η_F_	200 Free (10 years): 1.08 ± 0.08 m·s^−1^ 200 Free (11 years): 1.16 ± 0.08 m·s^−1^ 200 Free (12 years): 1.21 ± 0.09 m·s^−1^ 200 Free (13 years): 1.23 ± 0.12 m·s^−1^	200 Free (11 years): 1.20 ± 0.12 m·s^−1^ 200 Free (12 years): 1.26 ± 0.08 m·s^−1^ 200 Free (13 years): 1.28 ± 0.07 m·s^−1^ 200 Free (14 years): 1.23 ± 0.12 m·s^−1^
Alshdokhi et al. (2020)	To quantify and compare the transfer of dryland strength gains to adolescent backstroke and freestyle swimming performance	Longitudinal (8 weeks)	BM, H, RH	SF, VJ, BJ, PC, LF_ext_, RF_ext_, LF_int_, RF_int_, BE	HR, RPE	50 Free CG: 43.93 ± 7.11 s 50 Free EG: 44.23 ± 10.27 s 50 Back CG: 49.58 ± 6.31 s 50 Back EG: 49.18 ± 7.00 s 100 Free CG: 104.60 ± 12.35 s 100 Free EG: 102.58 ± 21.72 s 100 Back CG: 119.48 ± 18.69 s 100 Back EG: 113.81 ± 22.02 s	50 Free CG: 42.78 ± 7.13 s 50 Free EG: 42.19 ± 10.23 s 50 Back CG: 47.87 ± 6.88 s 50 Back EG: 47.08 ± 7.41 s 100 Free CG: 102.98 ± 12.33 s 100 Free EG: 99.08 ± 22.32 s 100 Back CG: 118.01 ± 18.89 s 100 Back EG: 112.01 ± 21.77 s
Amaro et al. (2017)	To analyze the effects of a period of swim training alone (CG), a dryland SC program based on sets/repetitions (EG1), plus swim training alone or a dryland SandC program that focused on explosiveness plus swim training alone (EG2)	Longitudinal (10 weeks)	BM, H	MF, MMI, VJ, BT	n.a.	50 Free CG: 33.76 ± 3.14 s 50 Free EG1: 33.92 ± 1.47 s 50 Free EG2: 33.43 ± 2.83 s	50 Free CG: 33.64 ± 3.04 s 50 Free EG1: 34.02 ± 1.61 s 50 Free EG2: 31.65 ± 2.53 s
Barbosa et al. (2010)	To develop a model for young swimmers' performance based on biomechanical and energetic parameters	Cross-sectional	BM, H, FM	SL, SF, v	CV, SI, η_F_	200 Free: 156.80 ± 17.30 s	
Barbosa et al. (2014)	To develop a classification system for young talented swimmers based on kinematical, hydrodynamic, and anthropometrical characteristics	Cross-sectional	FSA	v, dv, dv/v, C_Da_	n.a.	100 Free: 71.30 ± 6.12 s	
Barbosa et al. (2015)	To compare swimming power output between boys and girls, and model the relationship between swimming power output and sprinting performance	Cross-sectional	BM, H, AS, FSA	SF, SL, SL/AS, v, dv, dv/v, D_a_, C_DA_, P_d_, P_k_, P_ext_	SI, η_F_	Boys 100 Free: 1.44 ± 0.16 m·s^−1^ Girls 100 Free: 1.30 ± 0.12 m·s^−1^	
Barbosa et al. (2019)	To compare the anthropometrics, biomechanics and energetics in young swimmers of different competitive levels	Cross-sectional	BM, H, AS, FSA	SF, SL, SL/AS, v, D_a_, C_DA_, P_d_, P_k_, P_ext_, E_tot_, F_r_, v_h_, R_e_	SI, η_F_, dv	100 Free Tier 1: 1.75 ± 0.07 m·s^−1^ 100 Free Tier 2: 1.53 ± 0.11 m·s^−1^ 100 Free Tier 3: 1.38 ± 0.13 m·s^−1^	
Bielec and Jurak (2019)	To describe the anthropometric characteristics of prepubescent swimmers and to determine the contribution of chosen anthropometric factors to sports achievements	Cross-sectional	H, HW, HL, AS, BM, BMI, BF	v	n.a.	n.a.	
Costa et al. (2011)	To track and analyze freestyle performance during elite-standard male swimmers' careers, from 12 to 18 years of age	Longitudinal (12 to 18 years-old)	n.a.	n.a.	n.a.	50 Free: Δ = 5.85 ± 2.66% 100 Free: Δ = 4.89 ± 2.70% 200 Free Δ = 5.54 ± 2.23% 400 Free: Δ = 5.47 ± 2.23% 800 Free: Δ = 5.74 ± 3.24% 1500 Free: Δ = 5.34 ± 2.69%	
de Mello Vitor and Böhme (2010)	To assess the relationship among anthropometric variables, specific physical conditioning, swimming techniques and 100 m Freestyle performance	Cross-sectional	BM, H, AS, HL, HW, FL, FW, Biacr B, Biiliac B, AS/H, Biacr B/Biiliac B, TS, SS, BF	SF, SL, SI	AnP, CV	100 Free: 1.46 ± 0.07 m·s^−1^	
Denadai et al. (2000)	To verify whether critical speed can be used as a non-invasive method for the determination of speed at a blood lactate concentration of 4 mmol·l^−1^	Cross-sectional	BM, H	v	CV, Bl, V4	Beginner CV: 0.78 ± 0.25 m·s^−1^ Beginner V4: 0.82 ± 0.09 m·s^−1^ Trained CV: 1.08 ± 0.4 m·s^−1^ Trained V4: 1.19 ± 0.11 m·s^−1^	
Duché et al. (1993)	To determine the influence of anthropometric and bio-energetic parameters on swimming performance	Cross-sectional	H, SH, BM, BF, Biacr B, Biiliac B, TSA, BA, ULL, AL, ForL	v	VO_2max_, AnP, MP_30_	50 Free: 40.60 ± 7.20 s 100 Free: 85.60 ± 14.70 s 200 Free: 187.70 ± 30.60 s 400 Free: 399.00 ± 78.50 s	
Ferraz et al. (2020)	To verifyassociations between the anthropometriccharacteristics of young swimmers ofdifferent genders and different competitive levels with sports performance in the 50m and400m freestyle races at different levels.	Cross-sectional	BM, H, BMI, AS, AS/H	SF, SL	SI	Boys (U12) 50 m Free: 33.20 ± 1.98 s Boys (U12) 400 m Free: 326.48 ± 16.94 s Girls (U13) 50 m Free: 34.48 ± 2.34 s Girls (U13) 400 m Free:330.75 ± 25.92 s Girls (U12) 50 m Free:36.52 ± 1.85 s Girls (U12) 400 m Free: 364.18 ± 26.36 s	
Ferreira et al. (2019)	To examine the physiological and biomechanical responses related to the 400m swimming performance	Longitudinal (11 weeks)	BM, H	SF, SL	SI, HR, Bl, Bg	400 Free: 444.40 ± 76.95 s	400 Free: 408.95 ± 61.40 s
Ferreira et al. (2021)	To describe the evolution of middle-distance swimming performancealong with physiological and biomechanical changes in young swimmers during a trainingseason including three macrocycles.	Longitudinal (45 weeks)	BM, H, BMI	SF, SL	SI, HR, Bl, Bg, RPE	400 Free: 432.37 ± 71.78 s	400 Free: 366.66 ± 47.70 s
Figueiredo et al. (2016)	To evaluate the determinants of front crawl swimming sprint performance	Cross-sectional	BM, H, AS, HL, HW, FL, FW	SF, SL, SL/AS, dv, IdC	CV, SI, η_F_	25 Free Cluster 1: 1.52 ± 0.16 m·s^−1^ 25 Free Cluster 2: 1.47 ± 0.17 m·s^−1^ 25 Free Cluster 3: 1.40 ± 0.15 m·s^−1^	
Garrido et al. (2010a)	To identify the dryland strength and power tests that can better associate with sprint swimming performance	Cross-sectional	BM, H	LE, BP, CMJ, BT, BR	n.a.	25 Free: 16.12 ± 0.67 s 50 Free: 35.21 ± 1.98 s	
Garrido et al. (2010b)	To examine the effects of combined dryland strength and aerobic swimming training for increasing upper and lower body strength, power and swimming performance	Longitudinal (8 weeks)	BM, H	D_a_, C_Da_, LE, BP, CMJ, BT, BR	n.a.	EG 25 Free: Δ =6.95% EG 50 Free: Δ =4.77%	
Geladas et al. (2005)	To examine the relationship between anthropometry, some physical capacity traits and sprint swimming performance	Cross-sectional	BM, BF, H, TUEL, HL, FL, CC, Biacr B,Biiliac B, AFlex, SFlex	HJ, HG	n.a.	Boys 100 Free: 65.52 ± 0.25 s Girls 100 Free: 68.10 ± 0.22 s	
Hue et al. (2013)	To investigate the anthropometric and physiological characteristics of young Guadeloupian competitive swimmers	Cross-sectional	BM, BF, H, AS, LL	CMJ, HL, Glide	eVO_2max_, MAV	Boys 15 Free: 10.25 ± 0.33 s Boys 400 Free: 363.75 ± 20.16 s Girls 15 Free: 10.63 ± 0.21 s Girls 400 Free: 359.25 ± 14.86 s	
Jürimäe et al. (2007)	To examine the influence of energy cost of swimming, anthropometrical, body composition, and technical parameters on swimming performance	Cross-sectional	BM, BF, BMI, BMM, H, FM, FFM, AS, TBMD, SBMD	SF, SL, v	SI, C_s_, VO_2_, ΔLa	400 Free: 401.50 ± 53.80 s	
Kjendlie et al. (2004a)	To investigate the differences in the energy cost at submaximal velocities in boys, and to study the differences in the energy cost at different size scaled submaximal velocities	Cross-sectional	BL, BM, BSA	Bu, Vol	C_s_, VO_2_	50 Free: 33.70 ± 2.90 s 100 Free: 75.10 ± 5.50 s	
Kjendlie and Stallman (2008)	To compare drag in swimming children, quantify technique using the technique drag index, anduse the Froude number to study whether children reach hull speed at maximal swim speed	Cross-sectional	BL, BM, BSA, H	R_e_, F_r_, D_a_, C_Da_, D_p_, C_Dp_, TDI, v	n.a.	25 Free: 1.42 ± 0.12 m·s^−1^	
Lätt et al. (2009a)	To examine the development of specific physical, physiological, and biomechanical parameters during swimmers' maturing and the influence of such parameters on swimming performance	Longitudinal (2years)	BM, BMI, BF, H, AS, FM, BMM, FFM, TBMD, SBMD	v, SF, SL	SI, C_s_, VO_2_,ΔLa	400 Free: 373.30 ± 53.50 s	400 Free: 351.50 ± 50.40 s
Lätt et al. (2009b)	To examine the development of anthropometrical, physiological, and biomechanical parameters during swimmers' maturing and the influence of such parameters on swimming performance	Longitudinal (2years)	BM, BMI, BF, H, AS, FM, BMM, FFM, TBMD, SBMD	v, SF, SL	SI, C_s_, VO_2_,ΔLa	400 Free: 373.90 ± 39.20 s	400 Free: 354.20 ± 34.40 s
Majid et al. (2019)	To recognize the effect of special exercises in the development of the rapid strength of the muscles of the legs and arms and the completion of the 50m breaststroke	Longitudinal	BM, H	AE, KFE	n.a.	50 Breast: 49.84 ± 5.51 s	50 Breast: 42.26 ± 2.73 s
Marinho et al. (2011)	To determine and analyze the anaerobic critical velocity comparing it with short distances performances in the four swimming techniques	Cross-sectional	BM, H	n.a.	AnCV	50 m Free: 1.45 ± 0.18 m·s^−1^ 100 m Free: 1.39 ± 0.17 m·s^−1^ 200 m Free: 1.29 ± 0.14 m·s^−1^ 50 m Fly: 1.36 ± 0.18 m·s^−1^ 100 m Fly: 1.23 ± 0.14 m·s^−1^ 200 m Fly: 1.08 ± 0.11 m·s^−1^ 50 m Back: 1.21 ± 0.09 m·s^−1^ 100 m Back: 1.17 ± 0.09 m·s^−1^ 200 m Back: 1.13 ± 0.09 m·s^−1^ 50 m Breast: 1.09 ± 0.16 m·s^−1^ 100 m Breast: 1.04 ± 0.13 m·s^−1^ 200 m Breast: 0.93 ± 0.11 m·s^−1^	
Marinho et al. (2020)	To understand the relationship between the coaches' demographics (academic degree, coaching level, training experience) in the applied training content and the swimmers' technical ability and performance.	Cross-sectional	BM, H, AS	v, dv, SL, R_e_, F_r_, C_Da_	SI, η_F_	100 m Free (Acad_level_1): 75.51 ± 10.02 s 100 m Free (Acad_level_2): 74.55 ± 9.56s 100 m Free (Acad_level_3): 73.62 ± 7.64s 100 m Free (Coach_level_1): 76.79 ± 11.27s 100 m Free (Coach_level_2): 75.06 ± 9.31s 100 m Free (Coach_level_3): 73.65 ± 8.43s 100 m Free (Exp_ ≤ 5): 75.44 ± 9.57 s 100 m Free (Exp_ > 5): 74.60 ± 9.54s	
Mezzaroba and Machado (2014)	To determine the influence of age, anthropometry, and distance on stroke parameters and performance	Cross-sectional	BM, BF, H, TUEL, TLEL	V, SF, SL	SI	10–11 years 100 m Free: 1.10 ± 0.17 m·s^−1^ 10–11 years 200 m Free: 1.02 ± 0.15 m·s^−1^ 10–11 years 400 m Free: 0.95 ± 0.14 m·s^−1^ 12–13 years 100 m Free: 1.28 ± 0.12 m·s^−1^ 12–13 years 200 m Free: 1.14 ± 0.12 m·s^−1^ 12–13 years 400 m Free: 1.07 ± 0.14 m·s^−1^	
Morais et al. (2012)	To develop a structural equation model for performance in young swimmers based on selected kinematic, anthropometric and hydrodynamic variables	Cross-sectional	BM, H, AS, HSA	SL, dv, D_a_	SI	Boys 100 Free: 78.33 ± 12.07 s Girls 100 Free: 85.25 ± 13.89 s Together 100 Free: 82.07 ± 12.96 s	
Morais et al. (2013a)	To analyze a gender and sports level effect, and sports level-gender interactions on anthropometrics, kinematics and energetics	Cross-sectional	BM, H, AS, TTSA, HSA, FSA	v, SL, SF, dv	SI, CV, η_F_	Swimmers were faster in Tier and performance decreased until Tier 4 (for boys only and girls only)	
Morais et al. (2013b)	To follow-up the stability of performance and its determinant factors (i.e., anthropometrics, kinematics, hydrodynamics and efficiency)	Longitudinal (one competitive season)	BM, H, AS, TTSA, HSA, FSA, CP	D_a_, C_Da_, v, SL, SF, dv	SI, η_F_	Performance improved significantly between the three evaluation moments (for boys and girls pooled together and individually)	
Morais et al. (2014a)	To model a latent growth curve of the performance and biomechanics	Longitudinal (one competitive season)	n.a.	D_a_, C_Da_, P_d_, SF, dv	η_F_	100 Free: 72.05 ± 5.33 s	100 Free: 66.13 ± 5.16 s
Morais et al. (2014b)	To assess the intra- and inter-individual variability of the performance and its determinant factors within and between seasons according to gender and skill level	Longitudinal (two competitive seasons)	BM, H, AS, TTSA, HSA, FSA, CP	D_a_, C_Da_, v, SL, SF, dv	SI, η_F_	Boys (high skill) 100 Free: Δ =13.39% Boys (average skill) 100 Free: Δ =27.80% Girls (high skill) 100 Free: Δ =7.77% Girls (average skill) 100 Free: Δ =17.85%	
Morais et al. (2015)	To apply a new method to identify, classify, and follow up swimmers, based on their performance and its determinant factors, and to analyze the swimmers'stability over a competitive season with that method	Longitudinal (one competitive season)	AS, CP	C_Da_, v, dv, SL	SI, η_F_	High skill 100 Free: 71.17 ± 5.91 s Average skill 100 Free: 77.57 ± 4.44 s Low skill 100 Free: 83.67 ± 5.11 s	High skill 100 Free: 61.63 ± 2.90 s Average skill 100 Free: 68.64 ± 3.36 s Low skill 100 Free: 73.43 ± 3.92 s
Morais et al. (2016)	To compute a confirmatory model for swimming performance based on anthropometrics, strength, power output, kinematics, and efficiency.	Cross-sectional	BM, H, AS	BT, v, P_d_	η_F_	100 Free: 74.25 ± 8.80 s	
Morais et al. (2017)	To test a performance-predictor model based on swimmers' biomechanical profile, relate the partial contribution of the main predictors with the training program over time, and analyze the time effect, sex effect, and time × sex interaction	Longitudinal (three competitive seasons)	BM, H, AS	SF, SL, v, dv	SI, η_F_	Boys 100 Free: 76.26 ± 7.00 s Girls 100 Free: 79.06 ± 6.77 s	Boys 100 Free: 60.08 ± 3.22 s Girls 100 Free: 68.06 ± 4.40 s
Morais et al. (2020a)	To analyze the variations in performance, anthropometrics, and biomechanics break to gather insights on the detraining process	Longitudinal (11 weeks)	BM, H, AS, TTSA, HSA, FSA	D_a_, C_Da_, v, SL, SF, dv, P_d_, P_k_, P_ext_, E_tot_, F_r_, v_h_, R_e_	SI, η_F_	Boys 100 Free: 68.53 ± 6.81 s Girls 100 Free: 75.07 ± 7.84 s	Boys 100 Free: 70.05 ± 5.84 s Girls 100 Free: 76.53 ± 6.44 s
Morais et al. (2020b)	To classify, identify and follow-up swimmers into sub-groups (clusters), according to the performance and its biomechanical determinants, and analyze the individualvariations of each swimmer	Longitudinal (two competitive seasons)	BM, H, AS, TTSA, HSA, FSA, CP	D_a_, C_Da_, v, SL, SF, dv, P_d_, P_k_, P_ext_	SI, η_F_	High skill 100 Free: 68.07 ± 6.62 s Average skill 100 Free: 73.14 ± 4.87 s Low skill 100 Free: 82.60 ± 4.18 s	High skill 100 Free: 61.46 ± 3.43 s Average skill 100 Free: 65.33 ± 2.97 s Low skill 100 Free: 70.09 ± 3.48 s
Moreira et al. (2014)	To analyze the effects of growth on swimmers' biomechanical profile	Longitudinal (10 weeks)	BM, H, AS, HSA, FSA	D_a_, C_Da_, v, SL, SF	SI, η_F_	Performance (swim speed) significantly increased	
Nevill et al. (2020)	To explore which key somatic and demographic characteristics are common to all swimmers and identify further characteristics that benefit only specific strokes	Cross-sectional	BM, H, AS, BF, SH, ULL, UAL, LAL, HL, LLL, TL, LL, FL, ARG, FG, WG, TG, Calf G, AG, Biacr B, Biiliac B	v	n.a.	Boys 100 Breast: 97.70 ± 13.50 s Girls 100 Breast: 95.40 ± 9.50 s Girls 100 Back: 79.50 ± 5.00 s	
Ozeker et al. (2020)	To examine the effect of dry-land training in addition to swimming training on girl's strength and swimming performance	Longitudinal (8 weeks)	n.a.	v, SFlexion, SAbd, EExt, EFlex, HExt, HAbd, KFE, SAdd	CV	50 Free CG: 45.71 ± 7.44 s 50 Free EG: 35.24 ± 2.57 s 400 Free CG: 514.07 ± 92.58 s 400 Free EG: 352.57 ± 23.79 s	50 Free CG: 45.65 ± 7.42 s 50 Free EG: 34.25 ± 2.39 s 400 Free CG: 513.04 ± 92.98 s 400 Free EG: 343.98 ± 22.10 s
Poujade et al. (2003)	To define the determining factors 400 m performance	Cross-sectional	BM, BF, H, AS, BSA	v	C_s_, VO_2_	400 m Free: 335.00 ± 10.00 s	
Poujade et al. (2002)	To measure the Cs and to examine the relationship between Cs and velocity, morphology and stroking parameters	Cross-sectional	BM, BF, H, BSA, HLift	SF, SL, v	C_s_, C_s_/SA, C_s_/SA, HL, VO_2_	400 m Free: 335.77 ± 9.77 s	
Saavedra et al. (2013)	To determine the volume of training, how it evolves and its relationship with performance	Cross-sectional	BM, H, SH, AS	n.a.	n.a.	n.a.	
Saavedra et al. (2010)	To analyze swimming performance by developing multivariate predictive modelsbased on a wide variety of assessments from a multidimensional perspective	Cross-sectional	BM, BF, BMI, H, SH, AS, HL, HW, FL, FW, Biacr B,Biiliac B, Bitroch B, KB, EB, WB, CG, AFG, GG, TG, LG, AS/H,Biacr B/H, CG/H, GG/H, SSS	HJ, HG, AFlex, SFlex, Glide, SF, SL, v	SRE, FB, PT, SandR, SR, Abd, FAH, SI	n.a.	
Sammoud et al. (2018)	To use allometric models to estimate the optimal body size, limb segment length, and girth and breadth ratios associated with 100-m breaststroke speed performance	Cross-sectional	APHV, BM, H, AS, SH, BF, FM, FFM, BMI, ULL, UAL, LAL, HL, LLL, TL, LL, FL, ARG, FG, WG, TG, Calf G, AG, Biacr B, Biiliac B	v	n.a.	Boys 100 Breast: 97.70 ± 13.40 s Girls 100 Breast: 95.40 ± 9.50 s	
Sammoud et al. (2019)	To examine the effects of plyometric jump program in combination with swimming compared with swimming only on proxies of muscle power	Longitudinal (8 weeks)	APHV, BM, H	CMJ, SLJ, 25 m KWP, 25 m Free WP, v	n.a.	CG 15 Free: 9.53 ± 0.80 s CG 25 Free: 17.17 ± 1.20 s CG 50 Free: 37.50 ± 2.80 s EG 15 Free: 10.10 ± 0.50 s EG 25 Free: 18.20 ± 0.90 s EG 50 Free: 40.00 ± 1.70 s	CG 15 Free: 9.30 ± 0.80 s CG 25 Free:16.90 ± 1.40 s CG 50 Free: 37.60 ± 4.00 s EG 15 Free: 9.60 ± 0.40 s EG 25 Free: 17.52 ± 0.70 s EG 50 Free: 39.10 ± 1.50 s
Sammoud et al. (2021)	To examine the effects of an 8-week plyometric jump training program on jump and sport-specific performances inprepubertal femaleswimmers	Longitudinal (8 weeks)	APHV, BM, H, BMI	CMJ, SLJ	n.a.	CG 25 Free: 18.35 ± 1.19 s CG 50 Free: 40.51 ± 3.10 s EG 25 Free: 19.27 ± 1.13 s EG 50 Free: 42.79 ± 2.65 s	CG 25 Free: 18.50 ± 0.17 s CG 50 Free: 40.94 ± 0.59 s EG 25 Free: 18.05 ± 0.15 s EG 50 Free: 41.08 ± 0.52 s
Seffrin et al. (2021)	To evaluate the characteristics of body, anthropometry, and neuromuscular fitness in young swimmers from 11 to 23 years old, and fit multiple regression models to verify which evaluated factors better explain performance in 100 and 400 m Freestyle	Cross-sectional	BM, LBM, H, AS, SH, ULL, LLL, FL, HL, TTSA, TW	CMJ, SJ, HG, AvgPext, AvgPflex, PText, PTflex, AvgPer, AvgPir, PTer, PTir	n.a.	Boys 100m Free: 84.73 ± 11.15 s Boys 400 m Free: 393.35 ± 62.93 s Girls 100m Free: 81.11 ± 8.45 s Girls 400 m Free: 376.65 ± 32.52 s	
Silva et al. (2012)	To characterize the backstroke swimming technique through the stroke parameters and the inter-arm coordination	Cross-sectional	BM, H, AS	v, SF, SL, SL/AS, IdC	SI	Boys 25 m Back: 1.18 ± 0.14 m·s^−1^ Girls 25 m Back: 1.06 ± 0.14 m·s^−1^	
Silva et al. (2013)	To characterize the front crawl technique by assessing the general biomechanical parameters and the inter-arm coordination	Cross-sectional	BM, H, AS	v, SF, SL, SL/AS, IdC	SI	Boys 25 m Free: 1.46 ± 0.12 m·s^−1^ Girls 25 m Free: 1.37 ± 0.18 m·s^−1^	
Staub et al. (2020b)	To explore how consistent career pathways develop among age group swimmers	Longitudinal (8 years)	n.a.	n.a.	n.a.	n.a.	
Staub et al. (2020a)	To investigate within-sport specialization and entry age in the careers of German age-group swimmers	Longitudinal (8 years)	n.a.	n.a.	n.a.	n.a.	
Tijani et al. (2019)	To investigate the relationship between anthropometrical and stroking parameters and their contribution to sprint swimming performance	Cross-sectional	BM, H, AS, AS/H, BMI, BF	v, SF, SL	SI	50 m Free: 31.27 ± 1.10s	
Tsalis et al. (2012)	To examine the physiological responses, the strokeparameter changes and the ability to sustain a velocity corresponding to critical velocity during interval swimming	Cross-sectional	BF, FM, LBM, S9	v, SF, SL	HR, CV, CSR, Bl	Children 50 m: 37.70 ± 1.50 s Young 50 m: 32.40 ± 1.30 s Adult 50 m: 31.10 ± 2.20 s Children 100 m: 85.70 ± 4.80 s Young 100 m: 71.50 ± 2.90 s Adult 100 m: 68.20 ± 3.60 s Children 200 m: 191.80 ± 10.40 s Young 200 m:157.90 ± 9.20 s Adult 200 m: 151.30 ± 5.60 s Children 400 m: 400.40 ± 18.9 s Young 400 m: 332.30 ± 23.00 s Adult 400 m: 315.20 ± 14.60 s	
Zarzeczny et al. (2013)	To find out if critical swim speed estimated on the basis of two distances (50 and 400 m) corresponds to the results obtained during a standard 12-minute swim test	Cross-sectional	BM, H	v	CV, HR rest, RR sys, RR diast	12 min test Free: 0.85 ± 0.03 m·s^−1^ 12 min test Breast: 0.73 ± 0.02 m·s^−1^	

*n.a.—not applicable (i.e., not reported)*.

*Free, freestyle; back, backstroke; breast, breaststroke; fly, butterfly; Acad_level, the academic level of coaches (1, bachelor; 2, master; 3, philosophy doctor); coach_level, the training level of coaches (1, level 1; 2, level 2; 3, level 3); Exp, training experience of coaches (≤ 5, equal or less than 5 years; > 5, more than 5 years); CG, control group; EG, experimental group; U13, under the 13-year level; U12, under the 12-year level*.

## Discussion

The aim of this study was to review the current body of work on the influence of determinant factors related to swimming technique and anthropometrics in the performance of young swimmers. It was recognized that the performance of young swimmers is not exclusively dependent on one or a small set of determinant factors related to swimming technique and anthropometrics. It is rather influenced by a multidisciplinary interaction of several determinant factors. Furthermore, these factors and their partial contribution to performance can change over time according to the training plan or designed periodization.

### Anthropometrics and Growth

Most studies (N = 55, ~93%) included in this review assessed the anthropometrics. Body dimensions are related to nature, i.e., genetically determined (Saavedra et al., 2010; Majid et al., 2019; Tijani et al., 2019). Researchers are prone to assess the anthropometrics of young swimmers of both sexes, because these features play one of the major roles in the swimming performance, kinematics, energetics, and efficiency (Geladas et al., 2005; Jürimäe et al., 2007; Lätt et al., 2009a), in addition to hydrodynamics (Kjendlie and Stallman, 2008; Barbosa et al., 2014). Cross-sectional studies showed that variables such as height (H), arm span (AS), and hand length (HL) are strongly and positively correlated to Freestyle sprint performance (i.e., 50 or 100 m) (Geladas et al., 2005; Morais et al., 2012; Bielec and Jurak, 2019). The same trend was verified in breaststroke, in which swimmers with longer upper-limb lengths and wider girths had a significant advantage (i.e., better performance in the 100 m) (Sammoud et al., 2018). In backstroke (25- and 50-m pace), it was observed that postpubertal swimmers were significantly faster than their prepubertal counterparts (Silva et al., 2013). The significant higher body mass (BM), H, and AS shown by the postpubertal swimmers contributed to this (Silva et al., 2013). The same trend was verified in other freestyle distances (100, 200, and 400 m—Mezzaroba and Machado, 2014; 50 and 400 m—Ferraz et al., 2020), in which H, AS/H ratio (Ferraz et al., 2020) and other lengths related to upper- (TUEL) and lower-limbs (TLEL) lengths were significantly longer in mature swimmers (Mezzaroba and Machado, 2014).

Cluster analysis identifies homogeneous subgroups of swimmers within a larger sample (Barbosa et al., 2014; Morais et al., 2015, 2020b). Cluster analysis detects swimmers within a specific cluster that shares similar characteristics but is very different from other swimmers who do not belong to that cluster (Morais et al., 2015). Faster swimmers, competing in the 100-m freestyle, were clustered as a group with larger anthropometric features such as BM, AS, H, chest perimeter (CP), hand surface area (HSA), frontal surface area (FSA), trunk transverse surface area (TTSA), and body surface area (BSA) (Morais et al., 2015, 2020b). A study that aimed to identify key somatic variables in youth swimming recognized that all swimmers benefited from having less body fat (BF), wider shoulders and hips, longer AS, and forearm girth (FG) in the 100-m breaststroke and backstroke events (Nevill et al., 2020). This review only includes data related to breaststroke and backstroke from this article (Nevill et al., 2020) because only these strokes met the inclusion criteria (i.e., under 13 years of average age). Nonetheless, the authors agreed that such characteristics were common in the whole sample (over 13 years of average age), including the freestyle and butterfly strokes (Nevill et al., 2020).

As young swimmers grow until reaching full maturity, the best way to gather deeper insights into the influence of anthropometrics on swimming performance is to design longitudinal studies (Lätt et al., 2009a,b; Abbott et al., 2021). When following up over a competitive season, swimmers who achieved better performances (in the 100-m freestyle) also had larger body sizes (Morais et al., 2020b). A similar trend was verified in the 400-m freestyle (Lätt et al., 2009a,b). Moreover, a 3-year study that recruited 91 swimmers from a TID program showed that the AS was a major cause of performance improvement (Morais et al., 2017). Nonetheless, it was argued that swimmers must “relearn” the stroke mechanics to better use the propelling limbs, whenever meaningful body changes happen, such as during growth spurts (Morais et al., 2017). This happens because, as mentioned earlier, anthropometry not only has a direct effect on the performance of swimmers but also holds a concurrent effect on other scientific domains related to swimming techniques (Tijani et al., 2019; Morais et al., 2020b). That is, longer lengths like H and AS are strongly related to longer stroke length (SL) (kinematics) (Silva et al., 2012; Morais et al., 2017); whereas, larger TTSA or BSA is strongly related to more drag (hydrodynamics) (Barbosa et al., 2014).

Young swimmers are prone to have several growth spurts within a competitive season (Abbott et al., 2021). Such spurts contribute to the improvement in several variables related to swimming technique (Morais et al., 2013b, 2015). It was shown that, even during detraining periods (i.e., training breaks) the performance impaired, but anthropometry was responsible for slowing down such impairment (Moreira et al., 2014; Morais et al., 2020a). That is, during an 11-week detraining period, the swimmers continued to grow up. Because they were taller at the end of the break, it allowed them to minimize the performance impairment (Morais et al., 2020a). This highlights the importance of a systematic and frequent assessment of the anthropometrics.

### Biomechanics

Biomechanics is related to swimming techniques, such as SL, stroke frequency (SF), stroke index (SI), and intra-cyclic variation of the swim speed (dv), which are part of the “nurture” process and the ones that better explain performance (Lätt et al., 2009a; Barbosa et al., 2010; Morais et al., 2012). Top-tier swimmers are faster, because of better SL, SF, Reynolds number (R_e_), Froude number (F_r_), and hull speed (V_h_) scores (Barbosa et al., 2019). Faster swimmers were also prone to have less dv (Barbosa et al., 2014; Figueiredo et al., 2016) and deliver more in-water mechanical power (Barbosa et al., 2015, 2019; Morais et al., 2020b). Thus, it seems that the fastest swimmers can promote smaller speed fluctuations (Barbosa et al., 2014) and produce more power concurrently (Barbosa et al., 2019; Morais et al., 2020b). It can be argued that in-water power is related to more dry-land strength. It has been shown that variables related to dry-land strength were correlated with sprint swimming (Garrido et al., 2010a; Seffrin et al., 2021) and middle-distance events (400-m freestyle—Seffrin et al., 2021). Moreover, the power to overcome drag can be explained by 94% of the dry-land strength (Morais et al., 2016). However, faster swimmers are also under more active drag (D_a_) and coefficient of active drag (C_Da_) (Barbosa et al., 2019). It should be noted that drag variables, such as D_a_, passive drag (D_p_), C_Da_, and coefficient of passive drag (C_Dp_), are highly dependent on velocity, TTSA, and BSA (Kjendlie and Stallman, 2008). Thus, bigger and faster swimmers are prone to be under more drag (Barbosa et al., 2014, 2019). Indeed, “matured” age-group swimmers performing freestyle (Silva et al., 2012) and backstroke (Silva et al., 2013) had higher stroke kinematics scores [namely swim speed (v) and SL]. Conversely, non-significant differences were found in the index of coordination (IdC) (i.e., motor control) between pre and postpubertal swimmers (Silva et al., 2012, 2013).

Longitudinal studies showed that variables related to biomechanics change significantly over time (Lätt et al., 2009a; Morais et al., 2015, 2020b). As aforementioned, young swimmers undergo growth and maturation processes that lead to changes in the swimming technique (Lätt et al., 2009a; Morais et al., 2017). They are prone to improve the kinematics and kinetics over long-term periods of time (Morais et al., 2017, 2020b). Nonetheless, in specific moments of a season, young swimmers may impair the stroke biomechanics (Morais et al., 2013b, 2014b). Despite the variations within the season, swimmers improved the stroke biomechanics when comparing the beginning and the end of the season. Longitudinal research also reported that swimmers cluster in groups with similar traits related to stroke biomechanics (Morais et al., 2015, 2020b). As far as the long term is concerned, i.e., during one or several competitive seasons, the variables that better characterize each group may change over time. Swimmers improve and impair the stroke biomechanics several times over one or more competitive seasons (Morais et al., 2015, 2020b). Notwithstanding, variations may not occur at the same time across all clusters (Morais et al., 2015, 2020b). Moreover, it has been shown that swimmers are also likely to change groups; that is, switching to another subgroup or performance level. A swimmer who is assigned to the top-tier subgroup may not remain in that subgroup. It is possible that, over the season, the swimmer may drop to a lower tier, and lower-tier swimmers can climb up to top-tier groups (Morais et al., 2020b). Performance levels are very dynamic over time, and swimmers can move to different tiers quite often. The shift is due to a concurrent change in the determinant factors underlying the performance, which, in turn, depend on the developmental training program they are under, as well as the rate of growth and maturation.

The relationship between the in-water training programs and swimming biomechanics can be better understood when internal and external training loads are monitored. However, few studies addressed this topic in developing programs for young swimmers (Garrido et al., 2010b; Saavedra et al., 2013; Morais et al., 2014a). High-training volumes during the first part of a season (with low intensity, including warm-up, recovery, and slow-pace drills) led to an improvement in performance (Morais et al., 2014a). The same authors (Morais et al., 2014a) evaluated a group of swimmers during a competitive season in four different moments. They achieved 59% of the final performance in the second evaluation moment and 99% in the third moment. Between the 3rd and 4th (final) moments, the swimmers improved by only 1%, with the SF as the main determinant (Morais et al., 2014a). Between the 3rd and 4th moments, the periodization included an increase in the aerobic power and aerobic capacity (Morais et al., 2014a). As their older counterparts, young swimmers increase SF whenever they want to reach faster speeds (Mezzaroba and Machado, 2014; Barbosa et al., 2019). The researchers noted that changes in performance are related to the type of training swimmers were undergoing at the time of each evaluation moment. Thus, coaches can use different training strategies for their periodization to reach previously outlined goals and avoid burnout.

Studies also aimed to understand the effect of dry-land strength on the performance of young swimmers (Sammoud et al., 2019, 2021; Ozeker et al., 2020). During an 8-week intervention (aerobic in-water training concurrently with dry-land strength), Garrido et al. (2010b) reported a trend in sprint performance improvement (25- and 50-m freestyle) due to strength training. This was confirmed in other sprint events (50- and 100-m freestyle and backstroke) (Alshdokhi et al., 2020). Swimmers assigned to the experimental group presented a larger increase in the selected variables compared with the control group (Alshdokhi et al., 2020). It was suggested that the improvement in dry-land strength resulted in better swimming performance. Others aimed to provide deeper insights into the effect of different types of dry-land strength and conditioning programs on sprint performance (50-m freestyle) (Amaro et al., 2017). It was noted that swimmers under explosiveness training (i.e., performing the repetition quickly) presented larger improvements in swimming speed compared with performing repetition/sets training (Amaro et al., 2017). The phenomenon of post-activation potentiation performance enhancement is defined as a voluntary dynamic force production after a short and acute bout of high-intensity voluntary exercise (Blazevich and Babault, 2019). A study used three 30-s post-activation potentiation protocols (10 min before competition) to understand its effect on the performance and stroke kinematics (Abbes et al., 2018). Authors verified that all protocols presented non-significant effects on the 50-m freestyle performance, SL, and SF. A follow-up study analyzed the effect of tethered swimming as post-activation potentiation in the 50-m freestyle performance and stroke kinematics (SL), and non-significant effects were observed (Abbes et al., 2020). Therefore, both studies suggest an unclear effect of post-activation potentiation performance enhancement on young swimmers.

### Energetics and Efficiency

Energetics and efficiency also play a role in the performance of young swimmers. That said, the energetic spartial contribution to the performance increases with age (Zacca et al., 2020). It has been observed that VO_2_ during submaximal swimming speeds is significantly lower in children than adults (Kjendlie et al., 2004a). A study that selected anthropometrics, kinematics, energetics, and efficiency as main outcomes demonstrated that the 100-m freestyle performance was predicted by anaerobic power (AnP), critical velocity (CV), and SI (as an efficiency proxy) (de Mello Vitor and Böhme, 2010).

The CV is a variable commonly used to assess the energetics of young swimmers (Denadai et al., 2000; Marinho et al., 2011; Zarzeczny et al., 2013). It is calculated based on the distance-time slope of several events or swimming distances (Dekerle et al., 2002). It is highly correlated with aerobic performance and, hence, used to control training intensities (Zarzeczny et al., 2013; Figueiredo et al., 2016). However, CV may underestimate swimming intensity corresponding to speed at a blood lactate concentration of 4 mmo·l^−1^ in swimmers aged 10 to 12 years old (Denadai et al., 2000). It was suggested that it relates, instead, to the intensity corresponding to the maximum steady state of lactate concentration (Denadai et al., 2000). The CV has a significantly direct effect on the 200-m freestyle (Barbosa et al., 2010) and can also provide a strong explanation in the shorter events performances, such as the 100-m freestyle (de Mello Vitor and Böhme, 2010). Swimmers with faster CV also delivered better performances in the 100-m freestyle (Morais et al., 2013a) and 25-m freestyle time trials (Figueiredo et al., 2016).

Besides the SI, researchers also selected the Froude efficiency (η_F_) as another energetic proxy (e.g., de Mello Vitor and Böhme, 2010; Morais et al., 2014a). The SI measures the ability of the swimmer to complete a given distance with a particular speed in the fewest possible number of strokes (Costill et al., 1985). The η_F_ estimates the amount of work or power used to translate the body in water (Zamparo et al., 2020). Both variables are straightforward and less time-consuming to compute compared with a direct measurement of other energetics variables (Figueiredo et al., 2016; Barbosa et al., 2019; Morais et al., 2020b). Larger SI and η_F_ are associated with better performance in short distances, as the 100-m freestyle and 25-m freestyle time trial. Indeed, the fastest swimmers distinguish themselves from others because they have a better CV, SI, and ηF (Morais et al., 2013a; Figueiredo et al., 2016; Barbosa et al., 2019). Moreover, it should be highlighted that the increase in SI and η_F_ is related to the technical training that young swimmers undergo (Morais et al., 2017).

For longer events, such as the 400-m freestyle, the VO_2max_ (Duché et al., 1993; Poujade et al., 2003) and the VO_2peak_ (Jürimäe et al., 2007) were the best predictors of swimming performance within a set of energetic variables. Hue et al. (2013) showed that the fastest swimmers in the 400-m freestyle event also had better VO_2max_ than their slower counterparts. When tested by the 5 × 300-m protocol, young swimmers improved their swimming economy as they got older based on lower heart rate (HR) variability (Tsalis et al., 2012). In mid-distance events, another variable monitored very frequently was the energy cost of swimming (C_s_), which increases with swimming speed (Poujade et al., 2002; Kjendlie et al., 2004a,b). Nonetheless, one study pointed out that kinematics (SL and SF), anthropometrics (body length—BL, BM, and BSA), and HL did not explain the C_S_ in young swimmers (Poujade et al., 2002). The authors suggested that underwater torque, technical ability, and maturation could be strong predictors. Another study reported that passive torque presented a significant linear relationship with absolute C_s_ in young swimmers (Kjendlie et al., 2004b). Overall, there is solid evidence that, for similar swimming speeds, young swimmers have more C_s_ than their older counterparts (Zamparo et al., 2000; Kjendlie et al., 2004a). Thus, the differences between young swimmers and their older counterparts in the economy are due to the less-technical ability of the former ones.

Longitudinal studies showed that an improvement in energetics (VO_2_ and ΔLa) allowed an enhancement in performance (Lätt et al., 2009a,b). These studies were mostly focused on the 400-m freestyle (i.e., middle distance) (Lätt et al., 2009a,b; Ferreira et al., 2019). A research group followed boys (Lätt et al., 2009a) and girls (Lätt et al., 2009b) during two competitive seasons. It was observed that the VO_2_ was among the best predictors of performances of both sexes. Others noted significant correlations between a set of energetic variables (i.e., Bl and Bg) in the 400-m freestyle performance (Ferreira et al., 2019). Nevertheless, SI (efficiency) was the best predictor of all the variables assessed (Lätt et al., 2009a,b), or the one that presented the highest correlation with performance (Ferreira et al., 2019). Additionally, it was suggested that the 400-m freestyle enhancement during a season was highly related to an increase in the SI, suggesting that, when swimmers are in this age group, coaches should prioritize technical development of the swimmers (Ferreira et al., 2021). That said, the authors indicated that, concurrently, with the technical enhancement, physiological variables are as important to optimize swimming performance in such middle-distance events (Ferreira et al., 2021). Thus, at early ages, training should focus on learning the proper swimming techniques (i.e., technical training).

Nonetheless, the same reasoning (i.e., importance of energetics/efficiency) can be claimed in shorter race events, at least based on research carried out in the 100-m freestyle (Morais et al., 2013b, 2014b). The η_F_ increased or at least was maintained over time (Morais et al., 2020b). Additionally, high skillful swimmers yielded larger efficiency over time compared with their slower counterparts (Morais et al., 2014b, 2015). The HR (as an energetic indicator) may also present an association with the energetics of swimmers in the 50-m, 100-m, (Alshdokhi et al., 2020), and 400-m freestyle (Ferreira et al., 2019). Both studies reported that training has a positive effect on HR of young swimmers. That is, swimmers decreased the HR, suggesting that, for the same task (50-m and 100-m—Alshdokhi et al., 2020; or 400-m freestyle—Ferreira et al., 2019), they required less effort, with improved performance. Therefore, it can be implied that, besides the middle-distance events (i.e., 400-m freestyle), energetics/efficiency also presents a strong contribution in shorter events (like the 50 and 100-m freestyle).

### Performance in a Long-Term Athlete Development (LTAD) Perspective

Longitudinal studies can also help to understand the evolution of swimming performance from childhood to adulthood (Costa et al., 2011; Staub et al., 2020a,b). This research is paramount to better explain how the growth pace of each swimmer affects the performance and its determinant factors (Durand-Bush and Salmela, 2002). As previously noted, the performance level is highly dynamic and depends upon growth and maturation spurts, as well as the development program the swimmer is under. Stability assessment allows the prediction of the future success of young swimmers by the estimation of the performance progression. Based on the analysis of 242 young swimmers (from 12 to 18 years old), a study observed that swimmers should display a 14–19% improvement from childhood to adulthood in all freestyle events to become part of an elite group (Costa et al., 2011). The same authors also pointed out that the age of 16 is when the ability to predict the adult competitive level increases considerably. Thus, one cannot “neglect” a swimmer who, at a given moment, is slower than his/her peers, because, the following year, he/she can become one of the best in his/her age group (Morais et al., 2015, 2020b).

A study explored how consistent career pathways are among age-group swimmers (Staub et al., 2020b). Swimmers with better FINA points at 11 years old (including events, strokes, and distances) were more likely to be ranked during more years over the analyzed time frame (8 years), but the correlation showed a weak effect (Staub et al., 2020b). The authors argued that young swimmers should get the chance to yield from LTAD programs and should not be selected only by their age-group performance level (Staub et al., 2020b). It was claimed that LTAD programs should bring awareness about this phenomenon, which requires advanced understanding from coaches and other practitioners (Lang and Light, 2010).

It has been recently reported that both nature (i.e., anthropometrics) and nurture (i.e., training—namely sports technique) are important to excel in youth swimming (Barbosa et al., 2019). The best performers among three subgroups of swimmers (subgroup #1: age-group national champions, national record holders or enrolled in talent ID programs) scored very well in variables related to both nature and nurture parameters. Conversely, swimmers in the subgroup #3 (racing at local competitions) were weaker in both dimensions, and swimmers in the subgroup #2 (racing at national competitions) showed weaknesses in nature-related factors (i.e., anthropometrics) but were reasonably good in nurture factors (i.e., training). The subgroup #2 profile shows the potential of swimmers who may be seen as less genetically predisposed, as a result of an effective developmental program (Barbosa et al., 2019; Marinho et al., 2020).

As far as LTAD is concerned, there is also an ongoing dialog about the potential negative effects of large volumes of training in young swimmers (Nugent et al., 2017). Many coaches combine assumptions based on their experience with evidence-based practice. Recently, Marinho et al. (2020) have reported that an improvement in academic degree, coaching level, and coaching experience of the coaches presented a positive and significant contribution to swimming efficiency and performance of young athletes. Swimmers under the guidance of a coach with a higher academic degree, coaching level, or more years of coaching experience were more efficient and, concurrently, delivered better performances (Marinho et al., 2020). As youth swimming training should be focused on technical training (Morais et al., 2012), coaches should be able to provide their athletes with training in key skills and abilities based on such technique determinants. Therefore, age-group coaches are advised to design training programs that are underpinned on high-level and cutting-edge evidence.

Another major topic within LTAD is early specialization (Larson et al., 2019; Staub et al., 2020a). Early specialization refers to young athletes who limit their childhood to a single sport, deliberating their training and development on a singular sport (Baker, 2003). It was claimed that early specialization might promote far more risks than benefits (Wiersma, 2000). Youth athletes can suffer from social isolation, overdependence, burnout, manipulation, injury, and compromise their growth and maturation (Malina, 2010). Conversely, an athlete who practices a set of skills with increased frequency and duration becomes more proficient in those skills than one who practices them periodically (Wiersma, 2000). In competitive swimming, there are four competitive swim strokes and one event combining all (medley), as well as several race distances. Thus, in swimming, a within specialization may occur whenever a swimmer chooses and develops at an early age a single stroke or distance (or a combination of more than one stroke or distance, or both combined) (Staub et al., 2020a). A study showed that greater diversification within the same sport is positively correlated with success at the age of 18 (Staub et al., 2020a). Thus, the younger a swimmer enters the top 100, more likely he/she is to reach a top-tier at the age of 18 (Staub et al., 2020a). This suggests that early specialization may not be the best pathway to ensure higher performance in adulthood. Additionally, Larson et al. (2019) showed that a set of markers related to early specialization was related to burnout or a dropout in youth swimming. However, it was suggested that early specialization in one event, stroke or distance could be a way for coaches to accomplish qualification times and promote rapid adolescent success at the expense of long-term elite success as adults (Staub et al., 2020a). As such, developmental programs should expose young swimmers to a broad range of events (distances and swimming strokes) and even, at early stages, to other aquatic and non-aquatic sports.

## Conclusions

Performance of young swimmers is characterized by a multifactorial, holistic, and dynamic phenomenon relying on several features from different scientific domains. Better performance has always been related to better swimming techniques. Concurrently, anthropometry (e.g., higher AS, H, and upper limbs) also plays an important role in performance. Swimmers with larger body dimensions are the fastest. This suggests that anthropometry (i.e., nature) and training (i.e., nurture) play key roles. The contribution of energetics and efficiency becomes more important as the swimmer gets older or whenever the swimming event becomes longer. Performance enhancement of young swimmers should rely on LTAD programs, always taking into consideration the growth spurt and the external training load of the swimmer. Coaches are advised to monitor the rate of growth of their athletes, since this can affect their performance. They should put more focus on improving swimming technique and less on the external training load.

## Data Availability Statement

The datasets presented in this article are not readily available because none. Requests to access the datasets should be directed to Jorge E. Morais, morais.jorgestrela@gmail.com.

## Author Contributions

JM, TB, PF, AS, and DM conceived and designed the study. JM, TB, and AS performed the search and data analysis. PF and DM performed the quality assessment. JM carried out the drafting of the manuscript. All authors reviewed the manuscript and approved the submitted version.

## Funding

This work is supported by national funds (FCT—Portuguese Foundation for Science and Technology) under the project UIBD/DTP/04045/2020.

## Conflict of Interest

The authors declare that the research was conducted in the absence of any commercial or financial relationships that could be construed as a potential conflict of interest. The reviewer NDG declared a shared affiliation, with no collaboration, with one of the authors, AS, to the handling editor at the time of the review.

## Publisher's Note

All claims expressed in this article are solely those of the authors and do not necessarily represent those of their affiliated organizations, or those of the publisher, the editors and the reviewers. Any product that may be evaluated in this article, or claim that may be made by its manufacturer, is not guaranteed or endorsed by the publisher.

## Acronyms

**Anthropometrics**
AFG: arm flexed girth
AG: ankle girth
AL: arm length
APHV: age at peak height velocity
ARG: arm relaxed girth
AS: arm span
AS/H: arm span / height index
BA: body area
BF: body fat
Biacr B: biacromial breadth
Biacr B/Biiliac B: biacromial breadth/biiliac breadth index
Biiliac B: biiliac breadth
Bitroch B: bitrochanteric breadth
Biacr B/H: biacromial breadth/height index
BL: body length
BM: body mass
BMM: bone mineral mass
BMI: body mass index
BSA: body surface area
Calf G: calf girth
CC: chest circumference
CG: chest girth
CG/H: chest girth/height index
CP: chest perimeter
EB: elbow breath
FG: forearm girth
ForL: forearm length
FM: whole body fat
FFM: fat free mass
FL: foot length
FSA: frontal surface area
FW: foot width
GG: gluteal girth
GG/H: gluteal girth/height index
H: height
HL: hand length
Hlift: hydrostatic lift
HSA: hand surface area
HW: hand width
KB: knee breadth
LAL: lower arm length
LBM: lean body mass
LG: leg girth
LL: leg length
LLL: lower limb length
PS: propelling size
RH: reach height
SBMD: spine bone mineral density
SH: sitting height
SS: subscapular skinfold
SSS: sum of six skinfolds
S9: sum of nine skinfolds
TBMD: total bone mineral density
TG: thigh girth
TL: thigh length
TLEL: total lower extremity length
TS: triciptal skinfold
TSA: thoracic section area
TTSA: trunk transverse surface area
TUEL: total upper extremity length
TW: trunk width
ULL: upper limb length
UAL: upper arm length
WB: wrist breadth
WG: wrist girth
**Biomechanics**
AE: arm extension
AFlex: ankle flexibility
AvgPext: average power extension
AvgPflex: average power flexion
AvgPer: average power external shoulder rotation
AvgPir: average power internal shoulder rotation
BE: back extension
BJ: broad jump
BP: bench press
BR: ball range
BT: ball throwing
Bu: buoyancy
C_DA_: coefficient of active drag
C_Dp_: coefficient of passive drag
CMJ: countermovement jump
D_a_: active drag
D_aF_: drag factor
D_e_: drag efficiency
D_p_: passive drag
dv: intra-cyclic variation of the swim speed
dv/v: intra-cyclic variation of the swim speed/swim speed index
EExt: elbow extension
EFlex: elbow flexion
E_tot_: total power input
F_r_: Froude number
Glide: gliding variables
HG: hand grip
HExt: hip extension
HAbd: hip abduction
HJ: horizontal jump
HL: hydrostatic lift
HS: hand slip
IdC: index of coordination
KFE: knee flexion/extension
LE: leg extension
LF_ext_: left forearm external rotation
LF_int_: left forearm internal rotation
MF: mean force
MMI: mean mechanical impulse
PC: pronated chin-ups
P_d_: power to overcome drag
P_k_: mechanical power to transfer kinetic energy to water
P_ext_: external mechanical power
PText: peak torque extension
PTflex: peak torque flexion
PTer: peak torque external shoulder rotation
PTir: peak torque internal shoulder rotation
R_e_: Reynolds number
RF_ext_: right forearm external rotation
RF_int_: right forearm internal rotation
SAbd: shoulder abduction
SAdd: scapular adduction
SF: stroke frequency
SFlex: shoulder flexibility
SFlexion: shoulder flexion
SL: stroke length
SL·pSL^–1^: stroke length normalized for anatomical potential stroke length
SLJ: standing long jump
SL/AS: stroke length/arm span index
TDI: technique drag index
v: swim speed
v_h_: hull speed
VJ: vertical jump
Vol: body volume
UT: underwater torque
25-m KWP: a 25-m kick without a push
25-m free WP: 25-m freestyle without a push
ΔCM-CV: distance between the center of mass and the center of volume
α_63_: body angle with the water line
**Energetics/efficiency**
Abd: abdominals test
AnCV: anaerobic critical velocity
AnP: anaerobic power
Bl: blood lactate
Bg: blood glucose
C_s_: energy cost of swimming
C_s_/SA: energy cost of swimming calculated per unit of surface area
C_s_/SA.HL: energy cost of swimming calculated per unit of surface area and hydrostatic lift
CSR: critical stroke rate
CV: critical velocity
FAH: flexed arms hang
FB: flamingo balance
eVO_2max_: estimated aerobic power
HR: heart rate HR
rest: resting heart rate
MP_30_: mean power in 30 s
MAV: maximal aerobic velocity
η_F_: Froude efficiency
PT: plate tapping
RR sys: resting systolic blood pressure
RR diast: resting diastolic blood pressure
RPE: rate of perceived exertion
SandR: sit and reach
SI: stroke index
SR: shuttle run
SRE: shuttle run endurance
V4: velocity corresponding to a blood lactate concentration of 4mmol·l^–1^
VO_2max_: maximal oxygen uptake
VO_2peak_: peak oxygen uptake
VO_2_: oxygen consumption
ΔLa: net increase of blood lactate
